# Trematode Cercariae from *Lymnaea gedrosiana* in the Caspian Sea Littoral in Iran: a one health concern

**DOI:** 10.3389/fmicb.2023.1222599

**Published:** 2023-07-14

**Authors:** Aida Vafae Eslahi, Armin Aligolzadeh, Majid Pirestani, Zahra Gharibi, Amir Abdoli, Kareem Hatam-Nahavandi, Behzad Bijani, Milad Badri, Jennifer K. Ketzis

**Affiliations:** ^1^Medical Microbiology Research Center, Qazvin University of Medical Sciences, Qazvin, Iran; ^2^Department of Pathobiology, Faculty of Veterinary Medicine, Urmia University, Urmia, Iran; ^3^Department of Parasitology, Faculty of Medical Sciences, Tarbiat Modares University, Tehran, Iran; ^4^Infectious and Tropical Diseases Research Center, Hormozgan Health Institute, Hormozgan University of Medical Sciences, Bandar Abbas, Iran; ^5^Zoonoses Research Center, Jahrom University of Medical Sciences, Jahrom, Iran; ^6^Department of Parasitology and Mycology, School of Medicine, Iranshahr University of Medical Sciences, Iranshahr, Iran; ^7^Department of Biomedical Sciences, School of Veterinary Medicine, Ross University, Basseterre, Saint Kitts and Nevis

**Keywords:** freshwater snails, prevalence, seasonality, zoonoses, *Lymnaea (Radix) auricularia*

## Abstract

**Introduction:**

*Lymnaea gedrosiana* snails are hosts to a variety of trematode cercaria of public and veterinary health importance. In Guilan Province, Iran, a region with a high level of fish and bird farming and wetlands important for migratory birds, little is known about the trematode cercaria from *L. gedrosiana*.

**Methods:**

From April 2020 to October 2021, six freshwater sites in Guilan Province were sampled for Lymnaeidae snails three times per season (spring, summer, autumn and winter). Snails were exposed to light and heat to induce cercaria shedding and shredded cercaria were identified morphologically and molecularly.

**Results:**

In total, 5,712 Lymnaeidae snails were collected of which 3,288 (57.6%) were identified to be *L. gedrosiana* with 54.3% containing trematode cercaria. Snail and cercaria recovery were highest in the spring and summer. Trematode cercaria identified included *Telorchis assula*, *Hypoderaeum conoideum*, *Apharyngostrigea pipientis*, *Sanguinicola cf. inermis*, *Opisthioglyphe ranae*, *Diplostomum pseudospathaceum*, and *Australapatemon burti*.

**Discussion:**

The four trematodes *D*. *pseudospathaceum*, *S. inermis*, *A. burti*, and *A. pipientis* have not been previously reported in Iran; all four of these can infect migratory birds. The most common cercaria found, *H. conoideum* (18.3% of the snails) is of zoonotic importance. The third most common cercaria found, *S. inermis* (10.0% of the snails) is detrimental to fish production. Given the importance of the wetlands in the region for wildlife and migratory birds as well as the number of fish and bird farms in the area, efforts to control *L. gedrosiana* snails are needed to protect wildlife and human health. In addition, monitoring programs should be implemented to identify and prevent introductions of new trematode species.

## 1. Introduction

Freshwater snails are important members of the fauna and contribute to the biodiversity, as they can be involved in the diet of both invertebrates and vertebrates (Ardpairin et al., 2022). However, they also serve as intermediate hosts for a wide range of trematode larvae of public health and veterinary importance with snail-borne parasitic diseases affecting people worldwide. Freshwater snails serve a direct role in distribution of parasitic trematodes larvae with the distribution of snails and parasitic diseases relatively correlated (Salahi-Moghaddam et al., 2011; Glöer and Pešić, 2012; Dodangeh et al., 2019). Thus, elimination or management of snail populations can be beneficial in the control and interruption of these diseases (Lu et al., 2018). Of the freshwater snails, those in the Lymnaeidae family (pond snails), which contains approximately 100 specie, have been associated with trematode infections that impact human and animal populations (Yakhchali et al., 2013). To develop control or elimination programs, examining snail populations, particularly those in the Lymnaeidae, is required to determine the origin of infection of definitive hosts and for revealing the trematode fauna in relevant areas (Imani-Baran et al., 2011).

In Iran, 73 species (34 genera representing 14 families) of freshwater snails have been identified, of which 9 species (from 6 families) have been reported to be infected with trematode larvae (Glöer and Pešić, 2012; Dodangeh et al., 2019). However, there are limited surveys documenting the prevalence and diversity of larval digenean trematode infection in lymnaeid snails in Iran. Of the species documented to date, *Lymnaea gedrosiana* (syn. *Lymnaea auricularia, Radix auricularia*) is among the seven species of lymnaeid snails reported from Iran with a broad distribution in the country. It is a freshwater snail that habitats aquatic basins with diverse environmental conditions (Yakhchali et al., 2013). In the current study we aimed to evaluate the prevalence and regional distribution of trematode cercariae in *L. gedrosiana* snails in the Caspian Sea littoral, Iran to provide a better understanding of the trematode species in this snail species and to provide information that can be used in future trematode control programs. *Lymnaea gedrosiana* is the dominant species in the region and was selected to be studied over other snail species due to its ability to be a host to trematodes that infect humans and animals.

## 2. Materials and methods

### 2.1. Study area

Guilan Province (also known as Gilan, Northern Iran) is situated in the south and south-west of the Caspian Sea littoral. It has a humid subtropical climate with abundant rainfall, vast water sources and dense forest (Nasrollahzadeh et al., 2011). The province has an approximate population of 2.5 million (2016 census) with the primary agricultural economic activity being rice production. Other important agricultural activities in the region include poultry farming and fish farming, with fish farming growing in importance (Salehi, 2007; Pouryazdankhah et al., 2019; Shahbazi et al., 2020).

### 2.2. Site selection and snail collection

**Figure 1:**
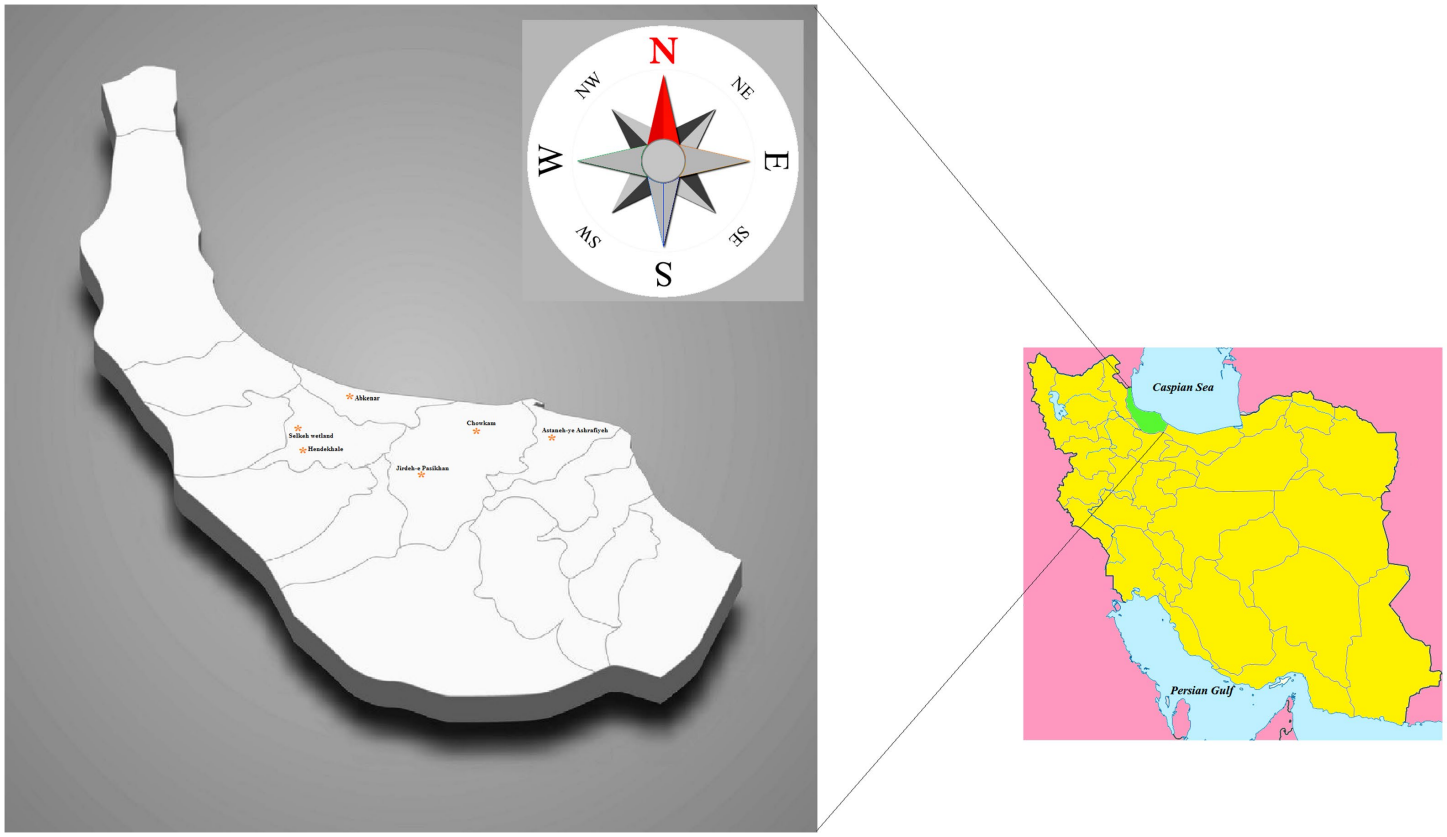
Map of Iran, geographical location of Guilan province. The raw map was downloaded from a free web source (https://commons.wikimedia.org/Category:Blank_maps_of_Iran) and edited by MB.

**Figure 2:**
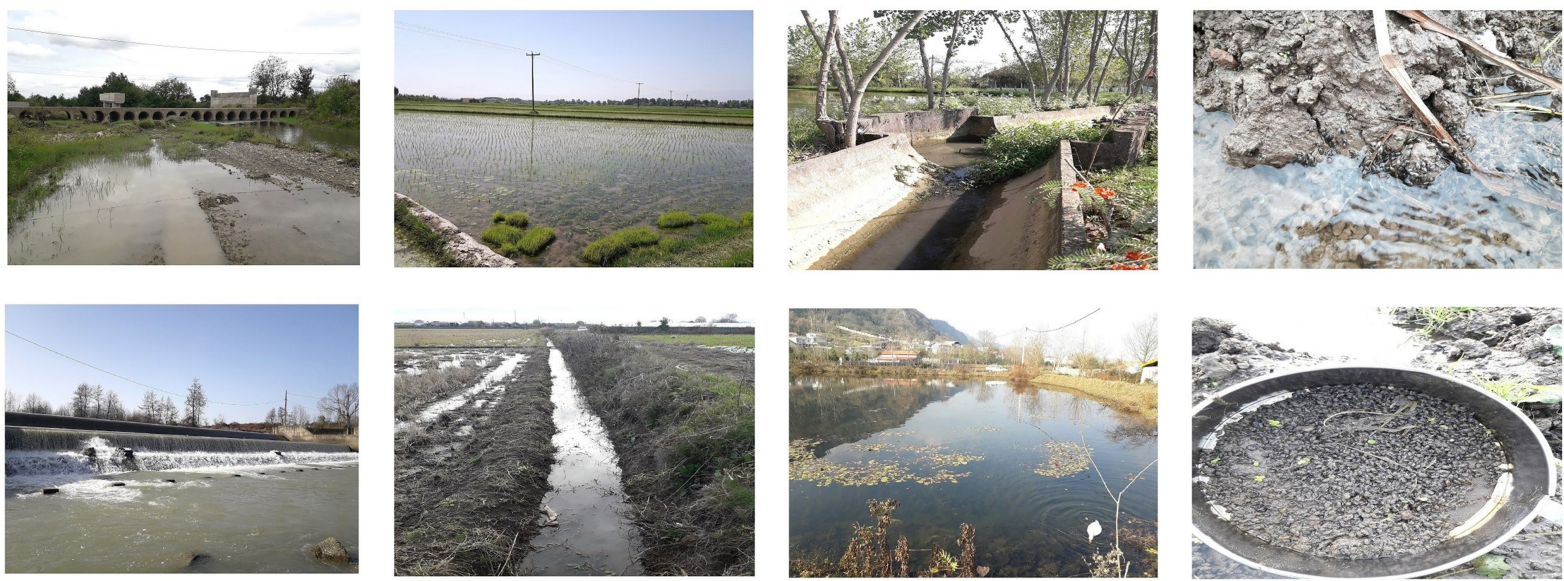
Examples of *Lymnaea gedrosiana* collection sites in Guilan province, Iran.

The present research was conducted from April 2020 to October 2021, inclusive. Six sites of freshwater snail habitats (near the villages: Chowkam, Abkenar, Selkeh, Astaneh-ye Ashrafiyeh, Jirdeh-e Pasikhan, and Hendekhale) in northern parts of Guilan Province located within the Caspian Sea littoral were monitored for *L. gedrosiana* (Figure 1). Each site was sampled three times (once per month) during each season with transects of the selected areas examined for snails. The snails were hand-picked or obtained using a scoop net from rice fields, irrigation canals, freshwater lakes, lagoons, reservoir, ponds, dams, and freshwater bodies near the livestock grazing areas (depth range 0–1.2 m; Figure 2). The collected snails were transferred to perforated plastic containers containing filtered pond water and transported to the Medical Microbiology Research Center, Qazvin University of Medical Sciences, Qazvin, Iran for identification. Recovered snails were identified using keys by Mansoorian (1986) and Pfleger (1998).

### 2.3. Cercarial shedding and cercaria identification

The snails were washed with de-chlorinated tap water and placed individually in glass Petri dishes filled with 50 mL de-chlorinated tap water. Shedding of cercariae was stimulated by exposing the snails to light and heat of a 100-W light bulb at a distance of 15 cm for 4–6 h. Afterwards, the water in each Petri dish was examined for the presence of cercariae using a compound microscope (X40 objective; Jayawardena et al., 2011). The morphological identification of cercariae was conducted using published keys (Frandsen and Christensen, 1984; Cichy and Żbikowska, 2016). To aid in identification, the recovered cercariae were stained with neutral red and Nile blue sulphate (vital staining) following published methods (Frandsen and Christensen, 1984). Avian schistosome species were excluded from the experiment due to another independent team conducting research on these species. Snails also were dissected and investigated for other developmental stages. Representatives of each type of cercaria was fixed with 96% ethanol and stored at-20°C until DNA extraction.

### 2.4. Molecular identification

DNA of isolates were extracted by FavorPrepTM Tissue Genomic DNA Extraction Mini Kit (FAVORGEN, Taiwan) according to the manufacturer’s instructions. The molecular identification was carried out *via* the 18S rRNA gene-based PCR test using the forward primer (18S 965) 5′-GGCGATCAGATACCGCCCTAGTT-3′ and the reverse primer (18S 1573R) 5′-TACAAAGGGCAGGGACGTAAT-3′ (Mullin et al., 2005). The PCR conditions involved an initial denaturation at 94°C for 5 min, 35 cycles of 94°C for 30 s, annealing at 57°C for 30 s, and extension at 72°C for 30 s, followed by a final extension at 72°C for 10 min. The PCR products were analyzed by gel electrophoresis using 1.5% agarose and separated with 80 amps for 50 min. PCR products were subsequently purified using the Cleanup PCR kit and directly sequenced by codon genetic group.^1^ The sequence data of each sample were confirmed using the standard nucleotide Basic Local Alignment Search Tool (BLAST) from the NCBI database.^2^ Consequently, the sequence data were aligned using the Muscle algorithm, and the phylogenetic tree was constructed with the MEGA7^®^ program. The analysis of data were performed with the maximum likelihood (ML) method based on the general time reversible (GTR) model with a discrete gamma distribution with invariant sites (G + I) following the Bayesian information criterion scores. The statistics for confirmed phylogenetic tree branches were evaluated using bootstrap resampling with 1,000 replicates.

## 3. Results

**Figure 3:**
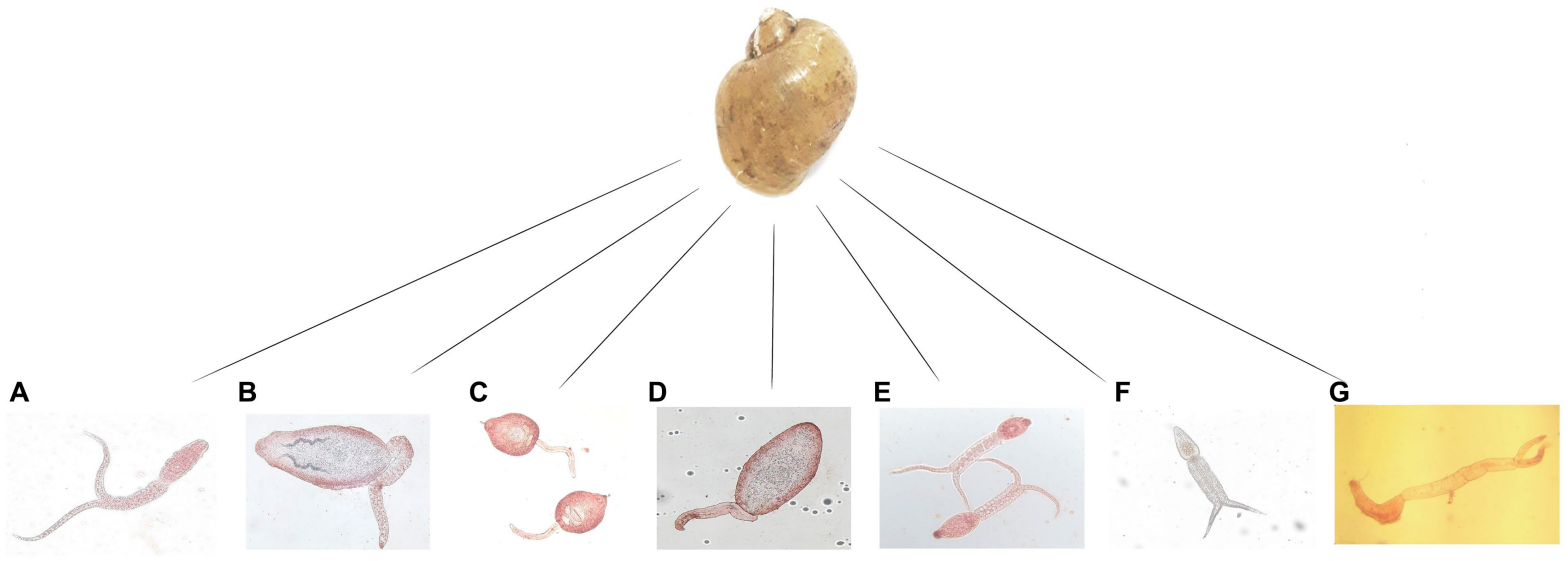
The seven types of cercariae recovered from *Lymnaea gedrosiana* in Guilan province, Iran. **(A)** furcocercariae, **(B)** echinostome cercariae, **(C,D)** xiphidiocercariae, **(E,F)** strigea cercariae and **(G)** lophocercous-apharyngeate cercariae,

In total, 5,712 Lymnaeidae snails were collected during the study period with 3,288 identified as *L. gedrosiana* (57.6%). The most *L. gedrosiana* were retrieved from Chowkam (635 snails), and Jirdeh-e Pasikhan (623 snails) with the least from Hendekhale (417 snails; Table 1). Cercariae infection from different trematodes was found in 54.3% of 3,288 examined snails (Table 1). Based on the study period, the majority of the infected snails were obtained between spring and summer (Table 2). There were seven different types of cercariae identified *via* morphological and molecular analyses. Five cercarial morphotypes were found: echinostome cercariae, xiphidiocercariae, furcocercariae, lophocercous-apharyngeate cercariae, and strigea cercariae (Figure 3). The most common trematode cercaria was echinostome cercariae (18.3%) and the least common was strigea cercariae (1.0%; Table 1).

**Table 1:** Geographical distribution of cercarial infection in examined *Lymnaea gedrosiana* snails of the Caspian Sea littoral of Iran.

Trematode (Cercarial morphotype)	Location						Total *N* = 3,288
	Chowkam *N* = 635	Abkenar *N* = 582	Selkeh *N* = 461	Astaneh-ye Ashrafiyeh *N* = 570	Jirdeh-e Pasikhan *N* = 623	Hendekhale *N* = 417	
	Number (% Positive)						
*Hypoderaeum conoideum* (echinostome cercariae)	260 (41.0)	181(31.1)	73 (15.8)	88 (15.4)	0	0	602 (18.3)
*Australapatemon burti* (strigea cercariae)	18 (2.8)	47 (8.1)	54 (11.7)	0	57 (9.1)	0	176 (5.3)
*Opisthioglyphe ranae* (xiphidiocercariae)	28 (4.4)	81 (13.9)	0	0	0	36 (8.6)	145 (4.4)
*Sanguinicola inermis* (lophocercous-apharyngeate cercariae)	83 (13.1)	0	0	117 (20.5)	129 (20.7)	0	329 (10.0)
*Telorchis assula* (xiphidiocercariae)	86 (13.5)	237 (40.7)	72 (15.6)	48 (8.4)	0	0	443 (13.4)
*Apharyngostrigea pipientis* (strigea cercariae)	0	34 (5.8)	0	0	0	0	34 (1.0)
*Diplostomum pseudospathaceum* (furcocercariae)	0	0	23 (5.0)	11 (1.9)	0	21 (5.0)	55 (1.6)
Total snails with cercaria	475 (74.8)	580 (99.7)	222 (48.2)	264 (46.3)	186 (29.9)	57 (13.7)	1,784 (54.3)

**Table 2:** Seasonal distribution of cercarial infection in examined *Lymnaea gedrosiana* snails of the Caspian Sea littoral of Iran.

Trematode (Cercarial morphotype)	Location	Season			
		Spring	Summer	Autumn	Winter
		No. positive/No. examined (%)			
*Hypoderaeum conoideum* (echinostome cercariae)	Chowkam	114/298 (38.2)	97/123 (78.9)	0/103	49/111 (41.1)
	Abkenar	87/221 (39.3)	56/163 (34.3)	38/107 (35.5)	0/91
	Selkeh	32/174 (18.3)	41/156 (26.2)	0/131	NA
	Astaneh-ye Ashrafiyeh	65/272 (23.8)	0/202	NA	23/96 (24.0)
	*Total*	298/965 (30.8)	194/644 (30.1)	38/341 (11.1)	72/298 (24.1)
*Australapatemon burti* (strigea cercariae)	Chowkam	6/298 (2.0)	0/123	12/103 (11.6)	0/111
	Abkenar	0/221	32/163 (19.6)	15/107 (14.0)	0/91
	Selkeh	30/174 (1.7)	0/156	24/131 (18.3)	NA
	Jirdeh-e Pasikhan	31/289 (10.7)	26/215 (12.0)	0/119	NA
	*Total*	67/982 (6.8)	58/657 (8.8)	51/460 (11.0)	0
*Opisthioglyphe ranae* (xiphidiocercariae)	Chowkam	0/298	0/123	28/103 (27.1)	0/111
	Abkenar	81/221 (36.6)	0/163	0/107	0/91
	Hendekhale	27/212 (12.7)	0/107	NA	9/98 (9.1)
	*Total*	108/731 (14.7)	0	28/210 (13.3)	9/300 (3.0)
*Sanguinicola inermis* (lophocercous-apharyngeate cercariae)	Chowkam	0/298	33/123 (26.8)	50/103 (48.5)	0/111
	Jirdeh-e Pasikhan	76/289 (26.2)	0/215	53/119 (44.5)	NA
	Astaneh-ye Ashrafiyeh	39/272 (14.3)	78/202 (38.6)	NA	0/96
	*Total*	115/859 (13.3)	111/540 (20.5)	103/222 (46.3)	0
*Telorchis assula* (xiphidiocercariae)	Chowkam	86/298 (28.8)	0/123	0/103	0 /111
	Abkenar	116/221 (52.4)	89/163 (54.6)	0/107	32/91 (35.1)
	Selkeh	0/174	72/156 (46.1)	0/131	NA
	Astaneh-ye Ashrafiyeh	0/272	48/202 (23.7)	NA	0/96
	*Total*	202/965 (20.9)	209/644 (32.4)	0	32/298 (10.7)
*Apharyngostrigea pipientis* (strigea cercariae)	Abkenar (Total)	34/221 (15.3)	0/163	0 /107	0/91
*Diplostomum pseudospathaceum* (furcocercariae)	Selkeh	23/174 (13.2)	0/156	0/131	NA
	Astaneh-ye Ashrafiyeh	11/272 (4.0)	0/202	NA	0/96
	Hendekhale	0/212	5/107 (4.6)	NA	0/98
	*Total*	34/658 (5.1)	5/465 (1.0)	0	0

The partial sequence data from PCR fragments of the 18S region were used to identify phylogenetic relationships. The length of these fragments was approximately 700–750 bp. The cercarial sequences were compared to sequence databases and the statistical significance was calculated by BLAST. The results showed that the sequences were aligned with *Telorchis assula* [Isolate 2019/Xiphidiocercaria] (100%), *Hypoderaeum conoideum* [isolate 1,266/Echinostome cercariae] (97%), *Apharyngostrigea pipientis* [isolate 904/Strigea cercariae] (100%), *Sanguinicola cf. inermis* [isolate 469/Lophocercous-Apharyngeate cercariae] (100%), *Opisthioglyphe ranae* [isolate 817/Xiphidiocercariae] (100%), *Diplostomum pseudospathaceum* [isolate 1,183/Furcocercariae] (100%), and *Australapatemon burti* [isolate 1,220/ Strigea cercariae] (100%).

**Figure 4:**
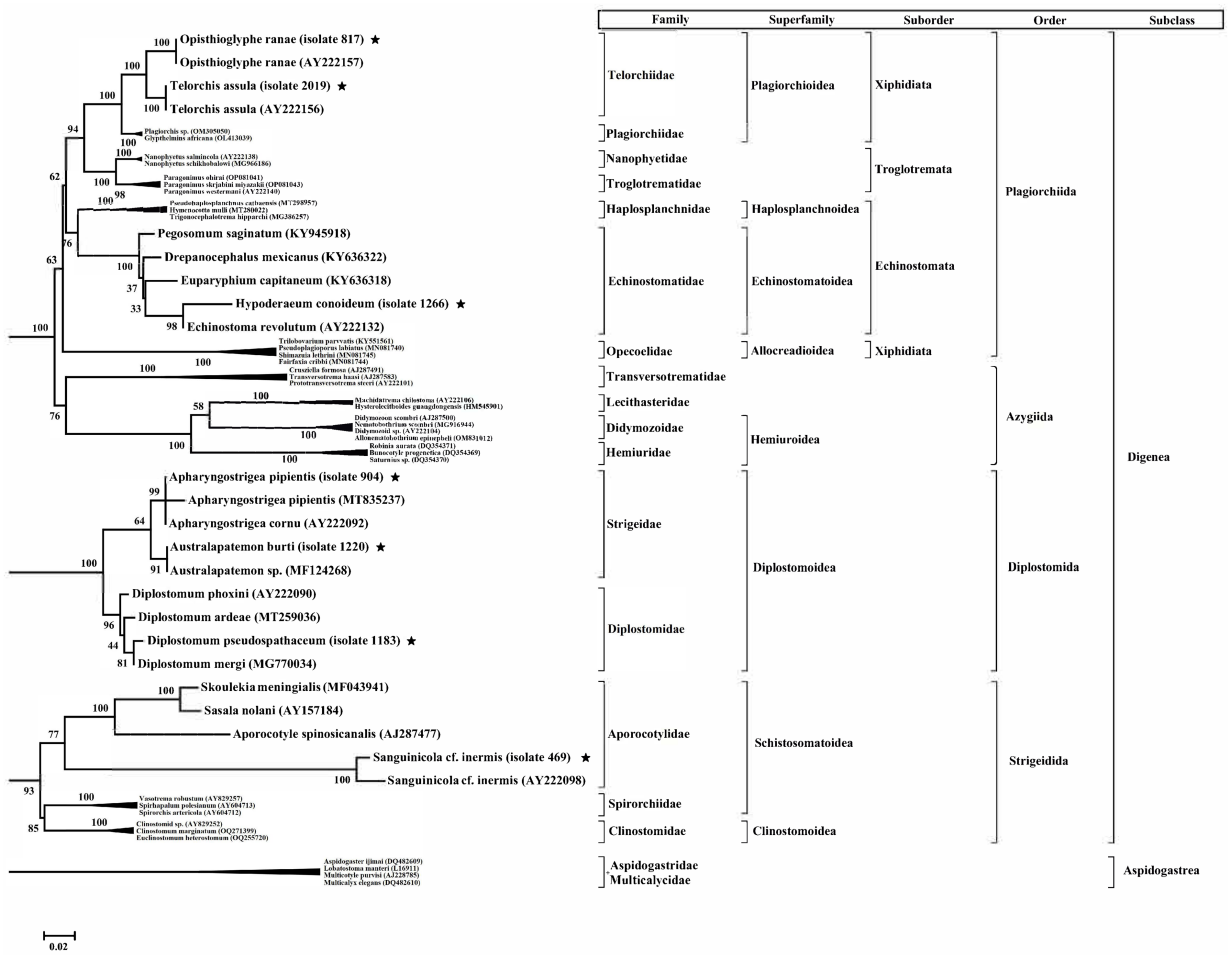
Midpoint-rooted tree of partial 18S rRNA sequences from 5 types of cercariae recovered from Lymnaea gedrosiana in the Caspian Sea littoral in Iran (indicated with an asterisk) and publicly available sequences from a range of related species of trematodes. The sequences of Aspidogastrean (Aspidogastridae + Multicalycidae) were used as an outgroup. Posterior probabilities are shown for most nodes, including all well-supported nodes. The taxonomic position of each species (subclass, order, suborder, superfamily, family) is shown on the right.

The phylogenetic tree of 18 s ribosomal RNA sequence data was constructed using maximum likelihood (ML) method with most suitable model, which the lowest Bayesian information criterion scores are considered to describe the substitution algorithm the best (GTR + G + I). All sequences appeared in a monophyletic tree using four sequences of Aspidogastrean as outgroup (Figure 4). This study revealed that the cercarial specimens can be divided into three clades according cercarial stage as well as separated from their classification. The first clade included two families of digenea (Telorchiidae/Xiphidiocercariae and Echinostomatidae/Echinostome cercaria). These were classified as Plagiorchioidea and Echinostomatoidea. The second clade showed that Strigeidae/Strigea cercariae and Diplostomidae/ Furcocercariae were classified as Diplostomoidea and the last group in this phylogenetic tree Aporocotylidae/Lophocercous-Apharyngeate cercariae classified as the Schistosomatoidea.

## 4. Discussion

*Lymnaea gedrosiana* are freshwater snails that are significant intermediate hosts for larval stages of trematodes with a vast distribution throughout freshwater environments of Iran (Moghaddam et al., 2004; Imani-Baran et al., 2010; Yakhchali, 2012; Dodangeh et al., 2019; Wiroonpan et al., 2022). Our findings revealed that *L. gedrosiana* was the most frequent species of lymnaeid snail in the investigated localities and each locality had its own digenean fauna. Also, a diversity of cercariae infecting *L. gedrosiana* snails were found in freshwater habitats of northern parts of Guilan Province with a relatively high prevalence (54.25%). To our knowledge, this is the first report of cercariae of the four trematodes *D. pseudospathaceum*, *S. inermis*, *A. burti*, and *A. pipientis* in Iran, indicating that the suitable climate conditions in Guilan province has an important role in the evolutionary cycle of these parasites. It also is, to our knowledge, the first molecular report of cercariae of the digenean trematodes in field-collected *L. gedrosiana* snails in the region.

In the study reported herein, there was a fluctuation in the number of snails in sampling sites with different ecosystems. Also, there was a seasonal variation in the abundance of snails, with higher numbers predominantly in spring and summer, which is in agreement with previous studies conducted on lymnaeid snails in Iran (Sharif et al., 2010; Imani-Baran et al., 2011, 2013). Several physicochemical and biological factors in the environment affect the distribution of snails in the freshwater ecosystem which can result in the seasonality observed. These factors are inclusive of temperature, characteristics related to water (current, turbidity, and transparency), water dissolution, dissemination of suspended solids, ion concentration, predator–prey interactions, competition, and food accessibility. For instance, it is indicated that climatic parameters such as rain and drought can limit the distribution of snails in most habitats (Okoye et al., 2022). In addition to physicochemical and biological factors, the presence of final hosts influences distribution.

Among the different sampling localities, Chowkam and Abkenar had the highest diversity in trematode species found and the highest cercaria infection rate in the snails. This is likely due to their location within the Anzali wetlands. These wetlands, a wildlife habitat and important aquatic ecosystem, serve as a nursery for many fish species that migrate to the area for spawning and are a breeding and wintering zone for a broad spectrum of aquatic birds from around the world. It is noteworthy that 77 of the 145 species of migratory birds in Iran, have been detected in Anzali wetland (Naderi et al., 2017; Khaleghizadeh et al., 2020; Ashrafi et al., 2021; Sadeghi Pasvisheh et al., 2021).

Identification and description of cercarial larvae *via* detailed morphological features is a traditional approach, which can be complemented with an investigation of intra-snail stages (Velázquez-Urrieta and de León, 2021). Molecular methods represent a reliable and precise detection of larval stages of trematodes (Li et al., 2022), making them effective complementary techniques to traditional methods detecting pathogens in intermediate hosts such as snails. The previous surveys on cercarial infection of *L. gedrosiana* in Iran have relied solely on morphology. In the current study we applied both morphological and molecular approaches and we could identify the cercariae of trematodes at the species level. According to the molecular characterization, the cercarial types belong to five families of digenean trematodes, i.e., Telorchiidae (*Telorchis assula* and *Opisthioglyphe ranae*), Echinostomatidae (*Hypoderaeum conoideum*), Strigeidae (*Apharyngostrigea pipientis* and *Australapatemon burti*), Diplostomidae (*Diplostomum pseudospathaceum*), and Aporocotylidae (*Sanguinicola* cf. *inermis*).

Some of the cercaria found in this study include trematodes of veterinary or public health importance including *H. conoideum* and *S. inermis*. *Hypoderaeum conoideum* is considered a neglected human-infecting trematode. It is among the 23 zoonotic species representing 8 genera of the Echinostomatidae family distributed mostly in Southeast Asia and Europe (Saijuntha et al., 2013; Chai, 2019). These trematodes are transmitted *via* ingestion of raw or inadequately cooked fish, amphibians, and molluscs and can cause abdominal pain, fatigue, diarrhea and weight loss in humans (Saijuntha et al., 2013; Chai, 2019). *Hypoderaeum conoideum* is commonly found in the small intestine of birds such as ducks, geese, swans, wild aquatic birds, and chickens in many regions of the world. Different species of freshwater snails serve as the first intermediate host, with fish, bivalves, and tadpoles having a role as the second intermediate host (Azizi et al., 2015; Yang et al., 2015). Within Guilan Province, breeding ducks and geese are very common and free-grazing ducks and geese are frequently rotated among different rice paddy fields within different areas of the province reflecting the significant role of these birds in the life cycle maintenance, distribution, and transmission of foodborne zoonotic parasites such as *H. conoideum* (Saijuntha et al., 2013). In addition to the ducks and geese, a high number of wild and migratory birds were observed during the snail collections, wandering around the sampling localities. Migratory birds likely play a major role in transmission of parasites such as *H. conoideum* serving as both final host and transport hosts (Youssefi et al., 2014). Between the suitable weather for snails and the high presence of birds, it can be concluded that Guilan is a favorable area for continuation of the *H. conoideum* life cycle.

Lophocercous-Apharyngeate cercariae of *S. inermis*, present in 10.0% of all the snails examined, is a blood fluke of freshwater cyprinid fish with severe pathogenic effects in the definitive fish host and significant impact on fish production (Richards et al., 1996; Kirk, 2012). It can cause serious economic problems in carp farms and mortalities in extensive fisheries (Kirk, 2012). Among the fish farming provinces in the country, Guilan has an important place in production of carp, a commonly sold and used fish in Iran, although carp used in farms are not native to the region (Salehi, 2007). Thus, the infection of snails with furcocercariae of *S. inermis* in the region must be taken in account in relevant surveillance sectors, especially the Iranian Fisheries Research Organization.

In our study, 13.4% of the sampled snails were recognized to be infected with *T. assula* cercariae. Different species of *Telorchis* inhabit the intestine of freshwater turtles, snakes, and salamanders. Snails are indicated to be among the contents in diet of both *Natrix natrix* and *N. tessellate* snakes (Al-Moussawi, 2015). In Iran, *T. assula* previously isolated from turtles (*Mauremys caspica caspica*), grass snakes (*N. natrix*), and dice snakes (*N. tessellate*) in north of the country (Yousesfi et al., 2013). These reptiles reside in wetlands where snails were sampled. There also was a high number of amphibians in most of the sampling localities, especially in spring and summer, which serve as hosts for *O. ranae*. The adult form of this trematode was formerly described in marsh frog (*Rana ridibunda ridibunda*) from Anzali Lagoon in Guilan (Mashaii et al., 2000).

*Diplostomum pseudospathaceum*, *A. burti* and *A. pipietins*, all of which have birds as final hosts, were also identified in our study. *Diplostomum pseudospathaceum*, the eye fluke of freshwater fish species, causes cataracts in the fish eye lenses decreasing visual ability. This can lead to increased predation of the fish by the final host for the trematode (Scharsack and Kalbe, 2014). Of the two Strigeidae trematodes identified, *A. burti,* which uses waterfowls, such as ducks and swans as a final host, was found more frequently (Aksenova et al., 2016). It has been reported from more than 11 different snail species from United States, Canada, and Europe (Calhoun et al., 2020). *Apharyngostrigea pipientis*, which uses wading birds as final hosts, are capable of forming their metacercariae around the pericardium of anuran tadpoles (Farrow, 2016). In the current study, infection of examined snails with furcocercous cercariae of *A. pipientis* could be connected with the presence of wetlands around the collection sites.

## 5. Conclusion

In the study presented herein, *L. gedrosiana* snails in the Caspian Sea littoral, Iran were found to be hosts to a variety of trematode cercaria. Only one identified trematode, *H. conoideum*, has a direct impact on human health; however, several other identified trematode species indirectly impact human health due to their negative impact on fish and bird production and all of them can impact wildlife, including migratory birds. Given the wide range of trematode cercaria that can use *L. gedrosiana* as a host and the importance of the wetlands in Guilan Province for migratory birds, a regular monitoring program of the cercaria in the snails should be introduced as well as *L. gedrosiana* control measures. Efforts to prevent the introduction of more trematode species, *via L. gedrosiana* control and cercaria monitoring, are needed to protect human and animal health within the region and more broadly.

## Data availability statement

The datasets presented in this study can be found in online repositories. The names of the repository/repositories and accession number(s) can be found at: https://www.ncbi.nlm.nih.gov/genbank/, OQ607702, OQ607703, OQ607704, OQ607705, OQ607706, OQ607707, OQ607708.

## Ethics statement

The animal study was reviewed and approved by the Ethics Committee for Research at Qazvin University of Medical Sciences.

## Author contributions

MB, ArA, and AE: designed the study. AE, ArA, AmA, BB, ZG, and MB: sample collection. AE, MP, and MB: experimental section. AE, MP, and MB: bioinformatic analysis. JK and MB: results interpretation and analysis. AE, KH-N, MB, and JK: manuscript writing. All authors contributed to the article and approved the submitted version.

## Funding

This work was supported by Medical Microbiology Research Center, Qazvin University of Medical Sciences, and Infectious and Tropical Diseases Research Center of Hormozgan University of Medical Sciences under the contract no. IR.QUMS.REC.1401.041.

## Conflict of interest

The authors declare that the research was conducted in the absence of any commercial or financial relationships that could be construed as a potential conflict of interest.

## Publisher’s note

All claims expressed in this article are solely those of the authors and do not necessarily represent those of their affiliated organizations, or those of the publisher, the editors and the reviewers. Any product that may be evaluated in this article, or claim that may be made by its manufacturer, is not guaranteed or endorsed by the publisher.

## Footnotes

^1^https://codongeneticgroup.com/

^2^http://blast.ncbi.nlm.nih.gov/
